# In vivo bundle‐specific anterior cruciate ligament length changes during overground walking

**DOI:** 10.1002/jeo2.70823

**Published:** 2026-06-23

**Authors:** Yutaka Fujita, Tomoharu Mochizuki, Kota Yamamoto, Keiichiro Someya, Keisuke Maeda, Tomohiko Hayashi, Toyohiko Hayashi, Shigeru Takagi, Noriaki Yamamoto, Hiroyuki Kawashima

**Affiliations:** ^1^ Department of Orthopaedic Surgery Niigata University Medical and Dental Hospital Niigata Japan; ^2^ Graduate School of Science and Technology Niigata University Niigata Japan; ^3^ Department of Orthopaedic Surgery Niigata Rehabilitation Hospital Niigata Japan; ^4^ Interdisciplinary Program of Biomedical Engineering, Assistive Technology, and Art and Sports Sciences, Faculty of Engineering Niigata University Niigata Japan; ^5^ Emeritus Niigata University Niigata Japan

**Keywords:** anterior cruciate ligament, anteromedial and posterolateral bundles, image‐integrated motion capture, in vivo biomechanics, overground walking

## Abstract

**Purpose:**

The purpose of this study was to characterise in vivo bundle‐specific anterior cruciate ligament (ACL) length changes during level overground walking and evaluate synchrony between the anteromedial (AM) and posterolateral (PL) bundles. We hypothesised that bundle length changes during overground walking would be phase‐dependent and that the AM and PL bundles would exhibit distinct but highly coordinated patterns throughout the gait cycle.

**Methods:**

Thirty healthy young adults underwent gait analysis during level overground walking at a self‐selected speed. An image‐integrated motion analysis system combining optical motion capture (250 Hz) and fluoroscopy‐based three‐dimensional bone registration was used to reconstruct femoral and tibial motion. ACL bundle lengths were defined as centroid‐to‐centroid distances between anatomically identified attachment regions. Bundle length waveforms were expressed as relative changes referenced to initial heel contact. Phase‐specific bundle length changes (Δ length) were analysed during stance and swing and within four stance subphases. Synchrony between bundles was assessed using Spearman's correlation analysis.

**Results:**

Both bundles demonstrated greater length changes during swing than stance. Mean AM bundle length change was 7.1 ± 1.9 mm during stance and 10.6 ± 3.6 mm during swing (*p* < 0.001), while mean PL bundle length change was 9.1 ± 2.4 and 14.8 ± 4.1 mm, respectively (*p* < 0.001). During stance, bundle length changes remained small from loading response to terminal stance and increased markedly during pre‐swing. The PL bundle showed greater geometric length‐change amplitude than the AM bundle across most gait phases, except terminal stance. AM and PL bundles exhibited highly synchronised length‐change waveforms throughout the gait cycle (Spearman's *ρ* = 0.95, *p* < 0.001).

**Conclusions:**

ACL bundle length changes during overground walking were phase‐dependent, with greater geometric length‐change amplitude in the PL than AM bundle and highly synchronised behaviour. These findings provide reference data for bundle‐specific ACL mechanics during gait.

**Level of Evidence:**

Level IV.

**Abbreviations**
2D: two‐dimensional
3D: three‐dimensional
ACL: anterior cruciate ligament
AM: anteromedial
CI: confidence interval
CT: computed tomography
CV: coefficient of variation
ICC: intraclass correlation coefficient
IQR: interquartile range
IRB: institutional review board
MDC: minimal detectable change
MRI: magnetic resonance imaging
PL: posterolateral
RLA: Rancho Los Amigos
SD: standard deviation

## INTRODUCTION

The anterior cruciate ligament (ACL) plays a central role in maintaining knee stability by resisting anterior tibial translation and controlling tibial rotation [5]. It consists of anteromedial (AM) and posterolateral (PL) bundles, which have distinct femoral and tibial attachment regions and exhibit different length‐change behaviour according to knee flexion angle [4, 18, 22]. Therefore, bundle‐specific analysis during fundamental movement tasks such as gait may provide more detailed insight into physiological ACL function than assessment of the ACL as a single structure, particularly in the context of anatomical ACL reconstruction, which aims to restore native ligament behaviour.

Despite advances in imaging technology, phase‐specific in vivo reference data for bundle‐specific ACL geometric behaviour during natural overground walking remain limited. Prior in vivo analyses using fluoroscopy or dynamic magnetic resonance imaging have shown that ACL length tends to increase near knee extension and decrease with greater flexion; however, these studies generally involved relatively small cohorts, varying temporal resolution or treadmill‐based walking conditions [12, 23]. Although overground and treadmill gait are broadly similar, some differences in lower‐limb kinematics and muscle activation have been reported [1, 17]. Therefore, bundle‐specific ACL length‐change patterns during overground gait warrant further investigation.

To address this need, we applied an image‐integrated motion analysis approach combining motion capture with fluoroscopy‐based three‐dimensional analysis [7, 16]. This hybrid system enables dense time‐series tracking of femoral and tibial bone positions and corresponding estimations of AM and PL bundle length throughout an entire overground gait cycle. In addition, the present study included 30 participants, which is relatively large for an in vivo dynamic imaging study of ACL behaviour [23].

Because walking is a common daily activity and a frequent target of postoperative functional recovery, clarifying bundle‐specific ACL geometric behaviour during overground gait is clinically and biomechanically relevant. The central research question of this study was whether AM and PL bundle geometric behaviour during natural overground walking exhibits distinct yet coordinated patterns when assessed with high temporal resolution. Therefore, the purpose of this study was to characterise high‐resolution, phase‐specific geometric length‐change patterns of the AM and PL bundles and to quantify the degree of synchrony between their length‐change waveforms. We hypothesised that ACL bundle length changes would be phase‐dependent and that the AM and PL bundles would exhibit distinct but coordinated patterns throughout the gait cycle.

## MATERIALS AND METHODS

### Ethics and participants

This in vivo study was approved by the institutional review board (IRB number: 2022‐0065), and all participants provided written informed consent prior to enrollment. 30 healthy adult volunteers (21 male, 9 female) were recruited (mean age 19.8 ± 0.9 years). All had no history of knee injury, surgery, or musculoskeletal disorders. A priori power analysis (*α* = 0.05, 1–*β* = 0.80) indicated that 18 knees were required to detect a large within‐subject effect (Cohen's *d_x_* ≈ 0.8) in ACL bundle elongation. The assumed effect size was based on Cohen's conventional definition of a large effect and was considered reasonable in light of prior in vivo ACL studies demonstrating clear bundle elongation and length‐change magnitudes during gait and dynamic knee motion [12, 23].

### Gait task and data collection

Gait analysis was performed on an indoor 12‐m level walkway (Figure S1A). Participants walked at a self‐selected comfortable pace, beginning several steps before and continuing several steps beyond the calibrated capture volume to ensure steady‐state gait within the analysis region. An optical motion‐capture system (VICON612, Vicon Motion Systems Ltd.) equipped with eight infrared cameras (sampling at 250 Hz) recorded the three‐dimensional trajectories of 34 reflective skin markers placed on the pelvis and lower limbs according to an anatomical lower‐limb marker protocol for image‐integrated gait analysis (Figure S1B) [13]. Specifically, thigh markers were placed at the greater trochanter, medial and lateral femoral epicondyles, and around the femoral shaft, while shank markers were placed at the medial and lateral tibial condyles, medial and lateral malleoli, fibular head, tibial tuberosity and around the tibial shaft. All markers were placed by the same experienced investigator. For 12 thigh markers and 10 shank (lower‐leg) markers, custom reflective markers incorporating a steel ball were used to allow identification of their two‐dimensional positions on subsequent biplanar X‐ray images. Ground‐reaction forces were recorded simultaneously using six 1.0 × 1.0‐m force plates installed flush with the walkway surface and arranged as a 2 × 3 array in two side‐by‐side columns within the capture region (Kistler Instruments, Winterthur, Switzerland). Because subjects may adjust their gait to place the first step on a force plate, the second step of the limb of interest within the force‐plate array was used for analysis in principle. Trials were accepted when steady‐state gait was maintained without obvious gait adjustment or targeting and when the analysed foot contact was fully captured on a single force plate. Trials in which the analysed step overlapped two adjacent force plates were excluded and repeated until five accepted trials were obtained from each participant.

The gait cycle was separated into stance and swing phases. The stance phase (initial contact to ipsilateral toe‐off) was subdivided into four standard subphases according to the Rancho Los Amigos (RLA) model: [14] loading response (initial contact to contralateral toe‐off), mid‐stance (contralateral toe‐off to ipsilateral heel rise), terminal stance (ipsilateral heel rise to contralateral initial contact) and pre‐swing (contralateral initial contact to ipsilateral toe‐off). The swing phase corresponded to the period during which the foot was not in contact with the ground.

### Bone model preparation and 2D–3D registration

Immediately after gait capture, biplanar radiographic images of the knee region were obtained using a previously reported image‐integrated motion analysis workflow [10, 13, 16]. Three‐dimensional bone models of the femur and tibia were reconstructed from each participant's CT data (slice thickness, 0.5–1.0 mm) using a CT‐based three‐dimensional planning software platform (ZedKnee, LEXI Co.). According to the institutional radiology department, the estimated effective radiation dose associated with the full‐length lower‐extremity CT protocol used in this study was approximately 5 mSv, and this imaging protocol was approved by the institutional review board. The three‐dimensional positions of the femur and tibia were reconstructed by aligning subject‐specific bone models to two‐dimensional marker positions on fluoroscopic images using a previously validated image‐registration technique. This image‐matching approach was originally introduced by Sato et al. [16] and later validated by Nishino et al. [13], who reported a mean translational error of approximately 0.5 mm and a rotational error of about 0.68° for knee‐joint kinematics, allowing for high‐accuracy reconstruction of the three‐dimensional spatial relationship between the femur and tibia, including their anteroposterior positioning. The technique has since been applied in clinical gait analyses using the Knee CAS system (LEXI Inc.), a radiography‐based three‐dimensional lower‐extremity alignment assessment system that uses 3D‐to‐2D image registration [10, 13, 16].

Prior laboratory evaluations of the VICON system have reported sub‐millimetre static accuracy and approximately 1–2 mm dynamic tracking accuracy under controlled conditions [9]. In the present study, the marker trajectories described above were processed within a previously described image‐integrated workflow [13], including smoothing with a fourth‐order zero‐lag Butterworth filter (7 Hz) and least‐squares–based estimation of femoral and tibial segment poses. Because small errors arising from soft‐tissue artefact and skin‐marker displacement can influence absolute bundle‐length estimation, absolute ACL length was not used as the primary variable of interest. Instead, bundle length change (Δ length) was analysed to enhance measurement robustness and to minimise the influence of soft‐tissue artefact, which is known to affect absolute distance measurements but has a reduced effect on frame‐to‐frame length change.

### ACL bundle length calculation

**Figure 1:**
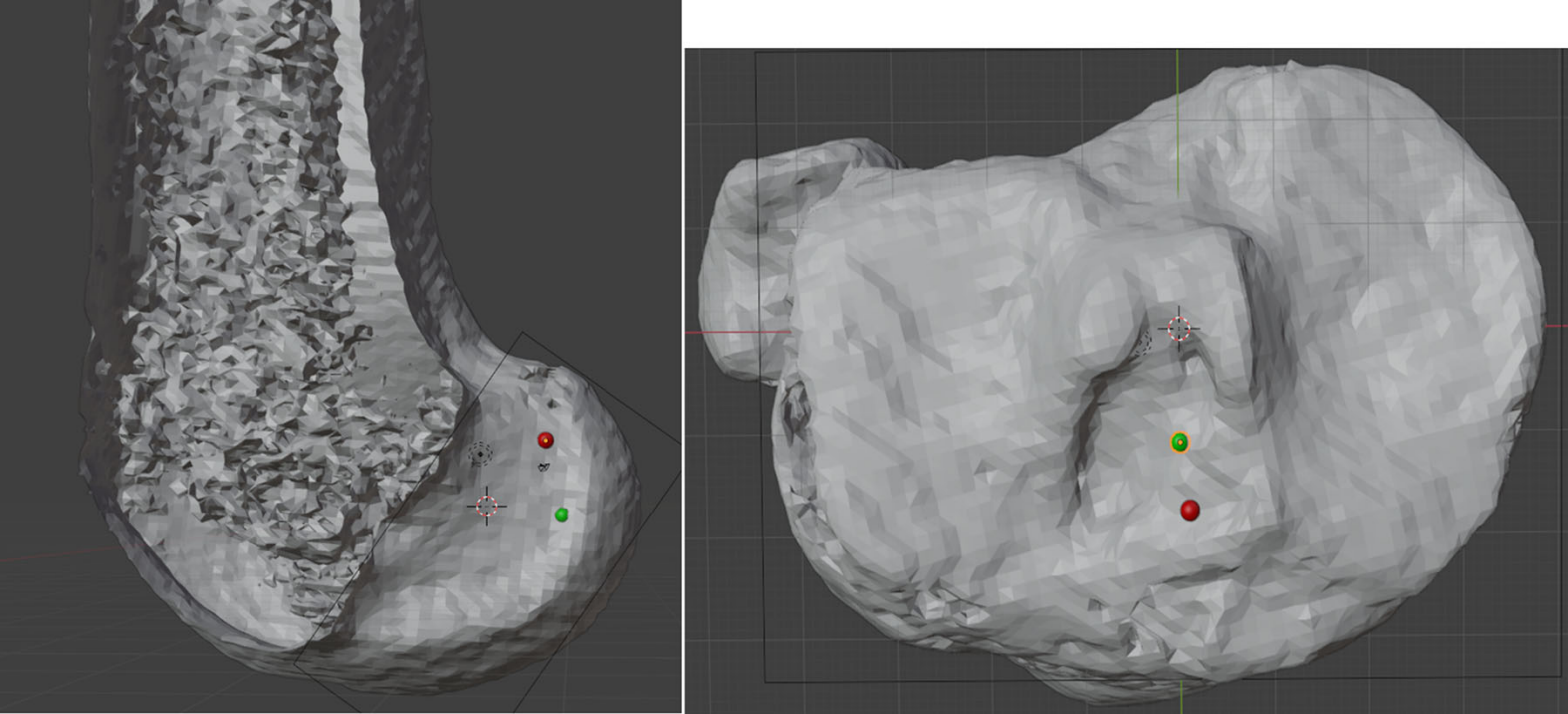
Three‐dimensional identification of the femoral and tibial attachment regions of the anteromedial (AM) and posterolateral (PL) bundles of the anterior cruciate ligament (ACL). The coloured markers indicate the centroids of the ACL bundle attachment regions: red markers represent the AM bundle attachment centroids, and green markers represent the PL bundle attachment centroids. Each femoral and tibial centroid was used as the representative insertion point for bundle length calculation throughout the gait cycle. The three‐dimensional bone models and attachment centroids were visualised using Blender software (Blender Foundation, Amsterdam, the Netherlands).

**Figure 2:**
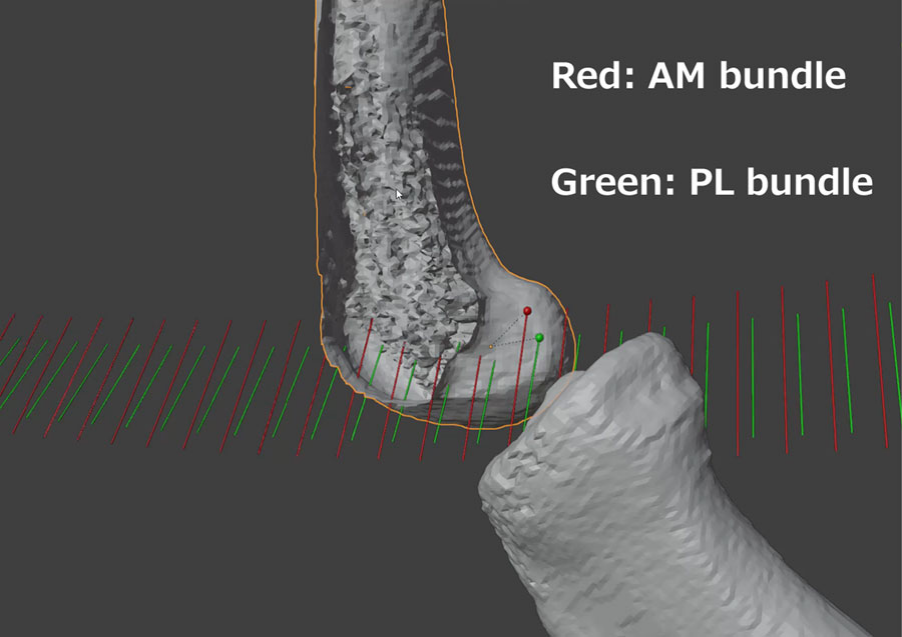
Three‐dimensional trajectories of the anteromedial (AM, red) and posterolateral (PL, green) bundle attachment centroids throughout the gait cycle. The paths illustrate the dynamic movement of the femoral–tibial insertion points during overground walking, providing the geometric basis for calculating instantaneous bundle length at each time point.

After registration, the femoral and tibial attachment areas of the ACL were identified on the reconstructed three‐dimensional bone models using Blender software (Blender Foundation, Amsterdam, the Netherlands), according to established AM and PL bundle regions described in previous anatomical studies [11, 15, 21]. Each attachment was divided into AM and PL regions, and the centroid of each region was used as the representative insertion point (Figure 1). Inter‐observer reliability of landmarking was assessed in 20 randomly selected knees. Two independent observers identified the femoral and tibial AM and PL centroids using the same anatomical criteria, and agreement was evaluated by intraclass correlation coefficients (ICC) based on the resulting bundle lengths. Because this centroid‐based measure represents a simplified geometric approximation rather than the true fibre length of the ACL, geometric bundle length in the present study was operationally defined as the Euclidean distance between the corresponding femoral and tibial centroids across the gait cycle (Figure 2). This centroid‐to‐centroid model was intended to represent region‐level geometric length change and does not capture fibre curvature, fanning, or intra‐bundle strain heterogeneity. Knee flexion angle was computed from the reconstructed three‐dimensional femoral and tibial bone poses obtained within the image‐integrated workflow, not directly from optical motion‐capture data alone, to describe knee motion throughout the gait cycle.

After geometric AM and PL bundle lengths were obtained throughout the gait cycle, length‐change magnitude (Δlength) was calculated for the stance and swing phases and for the four stance subphases: loading response, mid‐stance, terminal stance and pre‐swing.

Three types of comparisons were performed to examine ACL length change (ΔAM and ΔPL).

(1) Between the stance and swing phases, we tested whether the magnitude of geometric length change differed for each bundle (ΔAM and ΔPL).
(2) Within the stance phase, we compared ΔAM and ΔPL to determine whether there was any difference in their overall geometric length change across the stance period.
(3) Within each stance subphase, we evaluated ΔAM versus ΔPL.

To generate length‐change waveforms, instantaneous AM and PL geometric bundle lengths were calculated at each time point throughout the gait cycle. For each trial, the bundle length at initial heel contact was set as the reference value (0 mm), and the length change at each subsequent time point was calculated as the difference between the instantaneous bundle length and the initial‐contact length. Positive values indicated elongation relative to initial heel contact, whereas negative values indicated shortening. The resulting continuous length‐change data were time‐normalised from 0% to 100% of the gait cycle and used as the AM and PL bundle length‐change waveforms. For each participant, waveforms were averaged across five successful gait trials to generate representative curves. Group‐averaged waveforms were then used for the group‐level analysis, and subject‐level correlation coefficients were summarised across the cohort.

### Measurement reproducibility

To evaluate the reproducibility of this image‐integrated measurement approach, bundle length‐change measurements were assessed across repeated gait trials. Reproducibility was quantified using ICC, coefficients of variation (CV) and minimal detectable change (MDC) for each bundle across stance subphases and the overall stance and swing phases.

### Statistical analysis

Phase‐specific Δ length data are presented as mean ± standard deviation (SD) with 95% confidence intervals (CIs). For each participant, the five successful gait trials were first averaged to obtain participant‐level phase‐specific Δ length values. These participant‐level values were used for statistical analysis and for generating bar graphs. Bars represent group means, and error bars represent 95% CIs. Normality of the paired differences for each comparison was assessed using the Shapiro–Wilk test. Paired *t*‐tests were used for stance‐versus‐swing comparisons within each bundle and for AM‐versus‐PL comparisons within the overall stance and swing phases and each stance subphase. All tests were two‐sided, and statistical significance was set at *α* = 0.05.

To assess synchrony between the AM and PL bundles across the gait cycle, Spearman's rank correlation coefficient was calculated between the time‐normalised AM and PL length‐change waveforms from 0% to 100% of the gait cycle. Group‐level synchrony was calculated from the group‐averaged waveforms, whereas subject‐level synchrony was calculated from each participant's five‐trial‐averaged waveforms and summarised across the cohort as median and interquartile range (IQR).

Statistical analyses were performed using SPSS software (version 26.0; IBM Corp). Bar graphs were created using Python with the Matplotlib library.

## RESULTS

**Figure 3:**
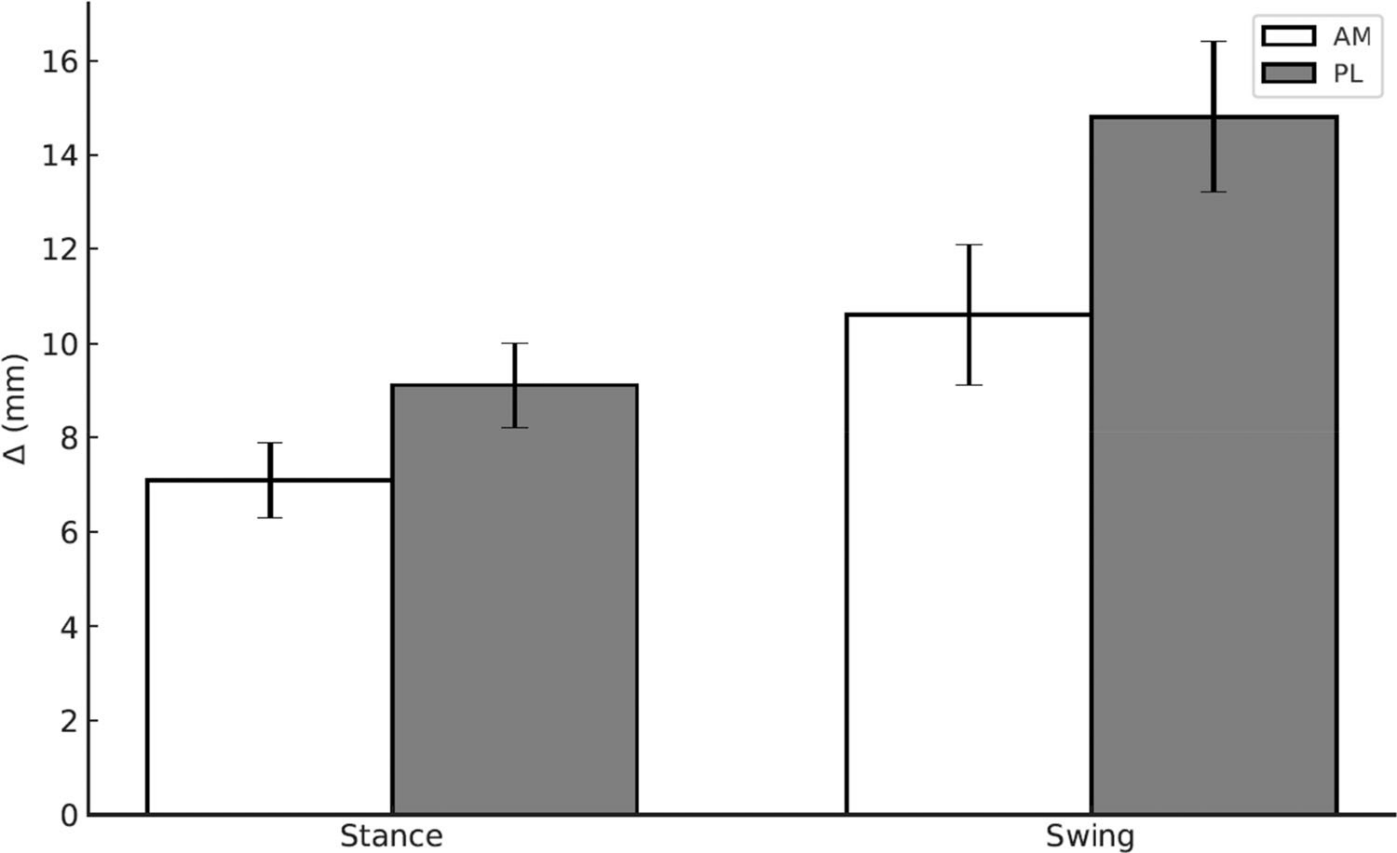
Bar graph showing anterior cruciate ligament (ACL) bundle length changes (Δ) of the anteromedial (AM) and posterolateral (PL) bundles during the stance and swing phases. Bars represent mean values, and error bars indicate 95% confidence intervals. The AM and PL bundles are displayed using distinct grayscale tones for clear differentiation.

During the swing phase, both bundles demonstrated larger geometric changes compared with the stance phase (Table 1): ΔAM = 10.6 ± 3.6 mm (95% CI, 9.2–12.1) and ΔPL = 14.8 ± 4.1 mm (95% CI, 13.3–16.4) (both *p* < 0.001). Within the stance phase, the mean change in length (Δ) of the AM bundle was 7.1 ± 1.9 mm (95% CI, 6.4–7.9), while that of the PL bundle was 9.1 ± 2.4 mm (95% CI, 8.2–10.0), with PL significantly greater than AM (*p* < 0.001) (Table 2, Figure 3). Relative to the AM bundle, the PL bundle demonstrated approximately 28% greater length‐change amplitude during stance and 40% greater excursion during swing.

**Table 1:** Comparison of ACL bundle length changes (Δ) between the stance and swing phases.

Bundle	Gait phase	Δ (mm), mean ± SD (95% CI)	*p*‐value (stance vs. swing)
AM bundle	Stance	7.1 ± 1.9 (6.4–7.9)	<0.001
	Swing	10.6 ± 3.6 (9.2–12.1)	
PL bundle	Stance	9.1 ± 2.4 (8.2–10.0)	<0.001
	Swing	14.8 ± 4.1 (13.3–16.4)	

*Note*: Both the anteromedial (AM) and posterolateral (PL) bundles showed significantly greater length changes during the swing phase than during the stance phase.

Abbreviations: ACL, anterior cruciate ligament; CI, confidence interval; SD, standard deviations.

**Table 2:** Comparison of ACL bundle length changes (Δ) between the anteromedial (AM) and posterolateral (PL) bundles within the stance and swing phases.

Gait phase	ΔAM (mm), mean ± SD (95% CI)	ΔPL (mm), mean ± SD (95% CI)	*p*‐value (AM vs. PL)
Stance phase	7.1 ± 1.9 (6.4–7.9)	9.1 ± 2.4 (8.2–10.0)	<0.001
Swing phase	10.6 ± 3.6 (9.2–12.1)	14.8 ± 4.1 (13.3–16.4)	<0.001

*Note*: In both phases, ΔPL was significantly greater than ΔAM.

Abbreviations: ACL, anterior cruciate ligament; CI, confidence interval; SD, standard deviations.

**Figure 4:**
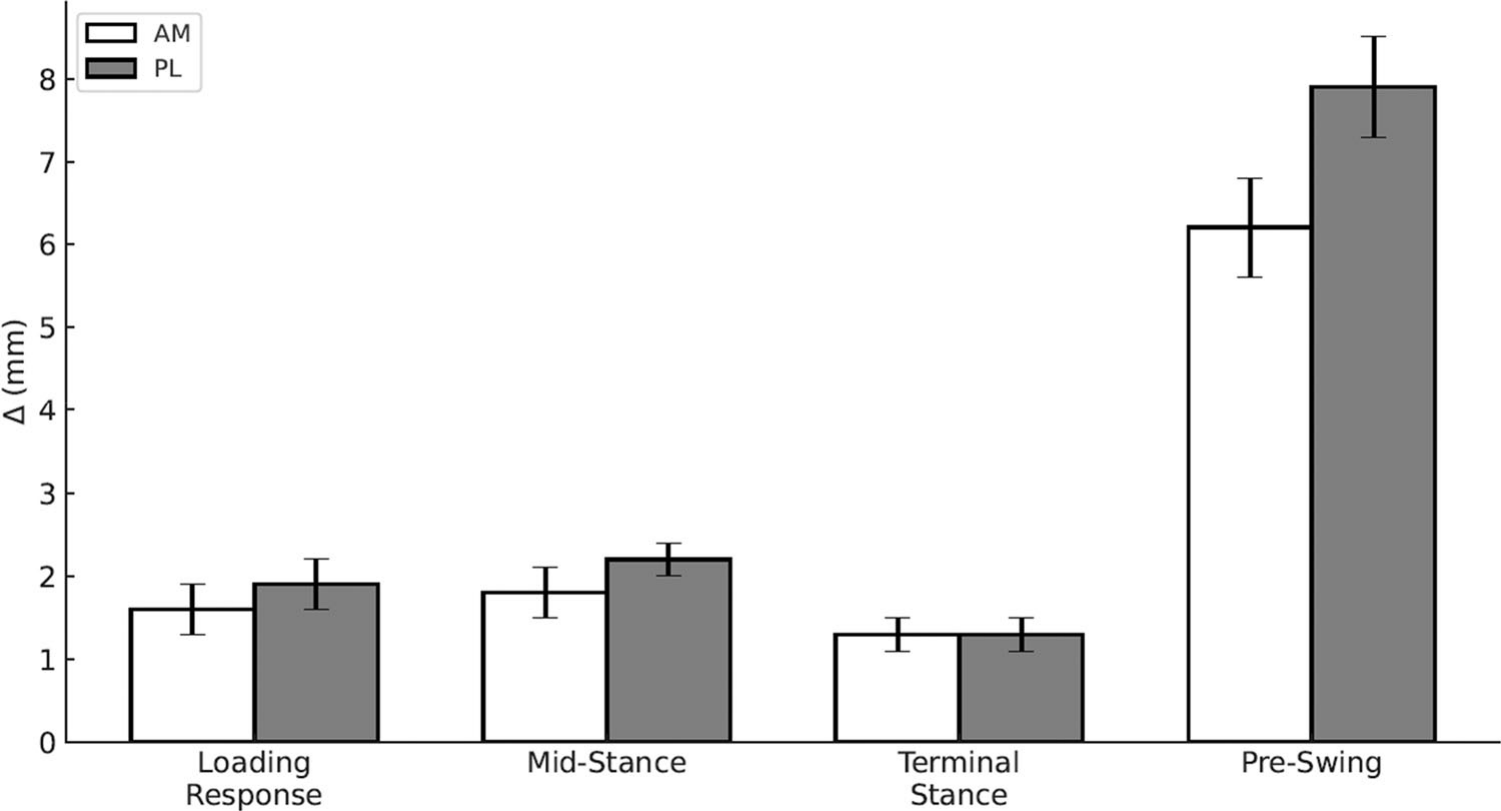
Bar graph illustrating anterior cruciate ligament (ACL) bundle length changes (Δ) for the anteromedial (AM) and posterolateral (PL) bundles across the four stance subphases: loading response, mid‐stance, terminal stance and pre‐swing. Bars represent mean values, and error bars denote 95% confidence intervals. AM and PL bundles are shown using distinct grayscale tones.

Subphase analysis of the stance phase revealed that ACL geometric length changes were minimal from loading response through terminal stance (Table 3, Figure 4). Specifically, ΔAM and ΔPL were 1.6 ± 0.7 mm (95% CI 1.3–1.9) versus 1.9 ± 0.7 mm (95% CI 1.6–2.2) in the loading response (*p* = 0.039). In mid‐stance, ΔAM and ΔPL were 1.8 ± 0.8 mm (95% CI 1.5–2.1) versus 2.2 ± 0.7 mm (95% CI 1.9–2.4), respectively (*p* < 0.001). In terminal stance, the changes were nearly identical between bundles (1.3 ± 0.6 mm [95% CI 1.1–1.5] vs. 1.3 ± 0.5 mm [95% CI 1.1–1.5]; *p* = 0.96). The smallest change was observed during terminal stance. In contrast, during the pre‐swing phase, ΔAM and ΔPL increased markedly to 6.2 ± 1.6 mm (95% CI 5.5–6.8) and 7.9 ± 1.6 mm (95% CI 7.2–8.5), respectively, with a significant difference between bundles (*p* < 0.001).

**Table 3:** Subphase‐specific comparison of anterior cruciate ligament bundle length changes (Δ) for the anteromedial (AM) and posterolateral (PL) bundles during the stance phase.

Stance subphase	ΔAM (mm), mean ± SD (95% CI)	ΔPL (mm), mean ± SD (95% CI)	*p*‐value (AM vs. PL)
Loading response	1.6 ± 0.7 (1.3–1.9)	1.9 ± 0.7 (1.6–2.2)	0.039
Mid‐stance	1.8 ± 0.8 (1.5–2.1)	2.2 ± 0.7 (1.9–2.4)	<0.001
Terminal stance	1.3 ± 0.6 (1.1–1.5)	1.3 ± 0.5 (1.1–1.5)	0.96
Pre‐swing	6.2 ± 1.6 (5.5–6.8)	7.9 ± 1.6 (7.2–8.5)	<0.001

*Note*: ΔPL exceeded ΔAM in loading response, mid‐stance and pre‐swing, while no difference was observed in terminal stance. Values are shown as mean ± SD with 95% confidence intervals (CIs).

**Figure 5:**
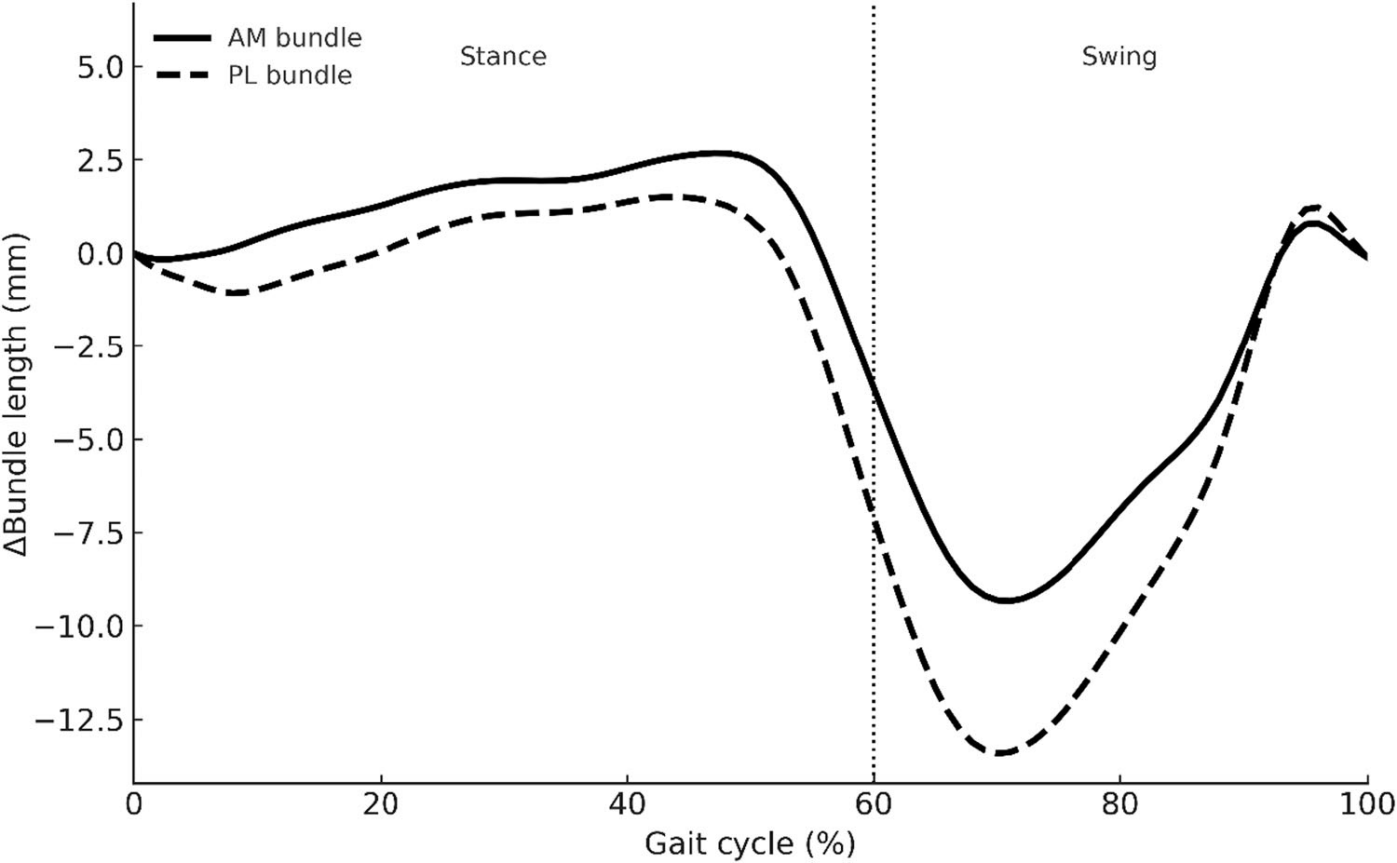
Mean geometric length‐change waveforms of the anterior cruciate ligament (ACL) bundles throughout the gait cycle. The solid line represents the anteromedial (AM) bundle, and the dashed line represents the posterolateral (PL) bundle. Bundle length‐change waveforms were generated by calculating the difference between the instantaneous geometric bundle length at each time‐normalised gait‐cycle point and the bundle length at initial heel contact, which was set as 0 mm. Positive values indicate elongation relative to initial heel contact, whereas negative values indicate shortening. The vertical dotted line at 60% indicates the transition from the stance phase to the swing phase. Each curve represents the group‐averaged waveform calculated from the participant‐level waveforms of 30 subjects.

A very strong correlation was observed between the group‐averaged AM and PL bundle geometric length–change waveforms (Spearman *ρ* = 0.95, *p* < 0.001), indicating that the elongation–shortening patterns of the two bundles were highly similar throughout the gait cycle (Figure 5). At the individual level, AM and PL bundle geometric length‐change waveforms showed consistently strong correlations across subjects, with a median Spearman's *ρ* of 0.95 [IQR, 0.93–0.96].

Intra‐trial reproducibility was excellent across all gait phases, with CVs of 2.5%−5.5%, ICCs of 0.88−0.99, and MDCs from 0.27 to 1.6 mm, indicating high measurement consistency across repeated trials and stance subphases (Table 4). Inter‐observer agreement for landmark‐based bundle length measurements was excellent, with ICC values of 0.90 for the AM bundle and 0.91 for the PL bundle.

**Table 4:** Reliability of V bundle length‐change measurements across stance subphases and full gait‐phase intervals.

Gait phase	Bundle	ICC	CV (%)	MDC (mm)
Loading response	AM	0.95	4.7	0.44
	PL	0.98	4.9	0.34
Mid‐stance	AM	0.97	4.9	0.32
	PL	0.94	2.5	0.41
Terminal stance	AM	0.95	5.5	0.27
	PL	0.88	5.0	0.39
Pre‐swing	AM	0.94	4.8	1.21
	PL	0.90	4	1.02
Stance phase (overall)	AM	0.92	4	1.6
	PL	0.92	3.6	1.8
Swing phase (overall)	AM	0.98	3.8	1.1
	PL	0.99	2.5	1.3

*Note*: Intraclass correlation coefficients (ICC), coefficients of variation (CV) and minimal detectable change (MDC) values are presented for both the anteromedial (AM) and posterolateral (PL) bundles. Subphase‐level reliability metrics are shown in the upper portion of the table, and overall stance and swing phase metrics are summarised in the lower portion.

## DISCUSSION

The most important finding of this study was that bundle‐specific ACL geometric length changes during overground walking were phase‐dependent yet highly coordinated. The AM and PL bundles showed very strong synchrony throughout the gait cycle, whereas the PL bundle demonstrated greater geometric length‐change amplitude than the AM bundle across most gait phases. These high‐resolution in vivo data quantitatively define phase‐specific bundle behaviour under physiologic overground conditions.

A methodological strength of this study is the integration of high‐frequency optical motion capture with fluoroscopy‐based 3D registration, which enabled evaluation of bundle‐specific ACL dynamics throughout the overground gait cycle in a relatively large in vivo cohort. Compared with previously reported dynamic imaging studies, the present system provided higher temporal resolution (250 Hz, approximately 1.7‐ to 8.3‐fold higher than conventional approaches) and included a larger sample size (*n* = 30, whereas most prior studies included fewer than 20 participants). This platform may be useful for future studies extending the analysis to pathological or reconstructed knees, thereby providing a framework for better interpreting clinical findings and symptoms in relation to altered ACL geometric behaviour.

During stance, particularly from loading response through terminal stance, geometric length changes remained small, which is consistent with previous studies showing that ACL length is influenced by knee flexion angle [6, 8, 20, 22]. Because knee flexion excursion is relatively limited during stance compared with the swing phase [20], within‐phase geometric ACL length change remained small during this period. This was most evident during terminal stance, when knee flexion angle changed least and both bundles showed minimal geometric length change, consistent with earlier imaging‐based observations [20, 23]. In contrast, rapid knee flexion during pre‐swing was associated with greater geometric length change in both bundles. However, these findings should be interpreted as geometric behaviour only, as geometric elongation does not directly indicate ligament strain or in situ loading.

The greater geometric length‐change amplitude observed in the PL bundle than in the AM bundle may be partly interpreted in relation to the known anatomical and functional differences between the two bundles. Previous anatomical and biomechanical studies have suggested that the AM bundle contributes to restraint over a broader range of knee flexion, whereas the PL bundle is relatively more taut near knee extension and becomes less taut with increasing flexion [5, 15]. In addition, intraoperative and biomechanical studies have shown that ACL graft length‐change patterns differ according to attachment or tunnel location during knee flexion–extension, with greater length changes reported for graft paths corresponding to the PL region than for those corresponding to the AM region [4, 19]. In this context, the smaller and more consistent geometric length change observed in the AM bundle may be compatible with its role in maintaining restraint across a wider flexion range. Conversely, the larger PL geometric length‐change amplitude observed in the present study may reflect greater sensitivity of the PL attachment region to flexion–extension‐related changes in femorotibial geometry during gait, which is also consistent with the concept that the PL bundle becomes relatively less taut with increasing knee flexion. However, because the present study quantified centroid‐to‐centroid geometric length change rather than true ligament strain or in situ force, these findings should be interpreted as anatomical and geometric associations rather than direct evidence of bundle‐specific loading.

Consistent with previous in vivo and dynamic imaging study reporting coordinated bundle behaviour during walking [3], the AM and PL bundles showed highly synchronised elongation–shortening waveforms throughout the gait cycle despite differences in geometric length‐change amplitude. This synchronised yet regionally differentiated pattern suggests that native ACL bundle behaviour during gait reflects coordinated responses to femorotibial motion, while the magnitude of geometric length change differs between regions according to bundle‐specific anatomy and attachment geometry. The waveform graph shows that relative geometric bundle length approached its maximum around terminal stance as the knee neared full extension, even though within‐phase change during terminal stance was small because knee motion in this phase was limited. This pattern suggests that terminal stance may be characterised by near‐maximal relative geometric bundle length with limited within‐phase geometric length‐change amplitude, rather than by substantial dynamic length change.

Although the overall temporal patterns were strongly synchronised, a very small phase‐specific deviation was observed. While synchronised motion was noted throughout most of the gait cycle, the PL bundle exhibited a brief, subtle shortening immediately after heel strike. This isolated deviation likely reflects transient unloading as the knee transitions from extension into slight flexion during weight acceptance—a position in which prior studies have reported reduced PL tension relative to the AM bundle [2, 5].

The present findings may have clinical relevance primarily as normative reference data rather than as direct guidance for rehabilitation restrictions. Specifically, although not directly prescriptive, the phase‐specific geometric elongation patterns observed in healthy knees may help interpret in vivo graft behaviour and provide a benchmark for assessing whether reconstructed bundles reproduce physiologic geometric elongation patterns during gait. In addition, the finding that the PL bundle showed greater geometric length‐change amplitude than the AM bundle across most gait phases may help interpret bundle‐specific graft behaviour after anatomic ACL reconstruction. However, because the present study quantified geometric length change rather than true ligament strain or in situ force, and because reconstructed grafts may not replicate native ACL behaviour, these findings should not be interpreted as direct evidence for modifying postoperative knee extension or weight‐bearing protocols.

### Limitations

Several limitations should be acknowledged. First, ACL length was defined as the centroid‐to‐centroid distance between femoral and tibial attachment regions, representing a simplified geometric approximation rather than true fibre length. This model does not account for fibre curvature, fanning, or intra‐bundle strain heterogeneity; therefore, the greater Δ length observed in the PL region should be interpreted as a region‐level geometric finding, not as direct evidence of greater mechanical tension or functional tautness. Because the true unloaded slack length of the ACL cannot be determined noninvasively in vivo, geometric elongation also does not directly translate to mechanical strain or in situ ligament force. In addition, electromyographic data were not collected, and the potential influence of neuromuscular activation could not be assessed.

Second, although the measurement framework integrated CT‐based 2D–3D registration with optical motion capture, soft‐tissue artefact cannot be completely eliminated. Despite the excellent reproducibility of relative length change, some degree of residual measurement uncertainty is unavoidable with any skin‐marker–based protocol.

Third, CT acquisition for three‐dimensional bone model construction inherently involved radiation exposure, which remains an important consideration in healthy volunteers. In addition, the technical complexity and time‐intensive setup of the integrated imaging system limited the number of participants, although the cohort was larger than in prior imaging‐based ACL studies.

Fourth, gait speed and other spatiotemporal gait parameters, such as step length and cadence, were not included in the present analysis. Although participants walked at a self‐selected comfortable speed under standardised overground conditions, the potential association between walking speed and ACL bundle length‐change patterns could not be evaluated. Future studies should incorporate spatiotemporal gait parameters to determine whether gait speed influences bundle‐specific ACL geometric behaviour.

Finally, only young healthy adults during level overground walking were included; the findings may not generalise to older individuals or pathological/postoperative ACL conditions. Additionally, the present analysis focused primarily on group‐level mean waveforms and phase‐specific averages, and inter‐subject variability patterns were not examined in detail. An important future direction will be to apply this methodology to reconstructed knees to evaluate whether bundle‐specific geometric length‐change patterns after reconstruction approach those of healthy knees. In addition, extension of this approach to higher‐demand tasks such as running, pivoting, stair navigation and jump landing will be necessary to more comprehensively characterise ACL function across a broader range of functional activities.

## CONCLUSION

This study quantified high‐resolution in vivo ACL bundle geometric behaviour during overground walking. Both bundles showed highly coordinated elongation–shortening patterns, with greater geometric length‐change amplitude during swing than stance and in the PL bundle than in the AM bundle. These findings define phase‐specific physiologic bundle behaviour under overground conditions and provide a quantitative benchmark for future comparisons with ACL‐deficient and reconstructed knees.

## AUTHOR CONTRIBUTIONS

Yutaka Fujita and Tomoharu Mochizuki contributed to the study conception and design. Yutaka Fujita performed data collection, data analysis and drafted the manuscript. Tomoharu Mochizuki contributed to methodology development, interpretation of the data, and critical revision of the manuscript. Kota Yamamoto, Keiichiro Someya and Keisuke Maeda contributed to data acquisition and technical support for motion analysis. Tomohiko Hayashi and Shigeru Takagi contributed to statistical analysis and interpretation of the results. Noriaki Yamamoto and Hiroyuki Kawashima supervised the study and provided overall project administration. All authors reviewed and approved the final manuscript.

## CONFLICT OF INTEREST STATEMENT

The authors declare no conflicts of interest.

## ETHICS STATEMENT

This study was approved by the Ethics Committee of Niigata University Medical and Dental Hospital (IRB No.2022‐0065). Informed consent was obtained from all individual participants included in the study.

## Supporting information

Supplementary Figure 1. Experimental setup for image‐integrated motion analysis. (A) Optical motion‐capture system with eight infrared cameras and six force plates embedded in a 12‐m level walkway. (B) Representative lower‐limb marker placement during overground walking. Reflective markers were attached to the pelvis and lower limbs, and custom reflective markers incorporating radiopaque steel balls were attached to the thigh and shank to allow identification on subsequent biplanar radiographic images.

Supporting File 1.

## Data Availability

The datasets generated during and/or analysed during the current study are available from the corresponding author on reasonable request.
