# Supplementary figures and images for: In vivo bundle‐specific anterior cruciate ligament length changes during overground walking
Source: J Exp Orthop. 2026 Jun 23;13(2):e70823. doi: 10.1002/jeo2.70823 (PMC13288363; doi:10.1002/jeo2.70823)

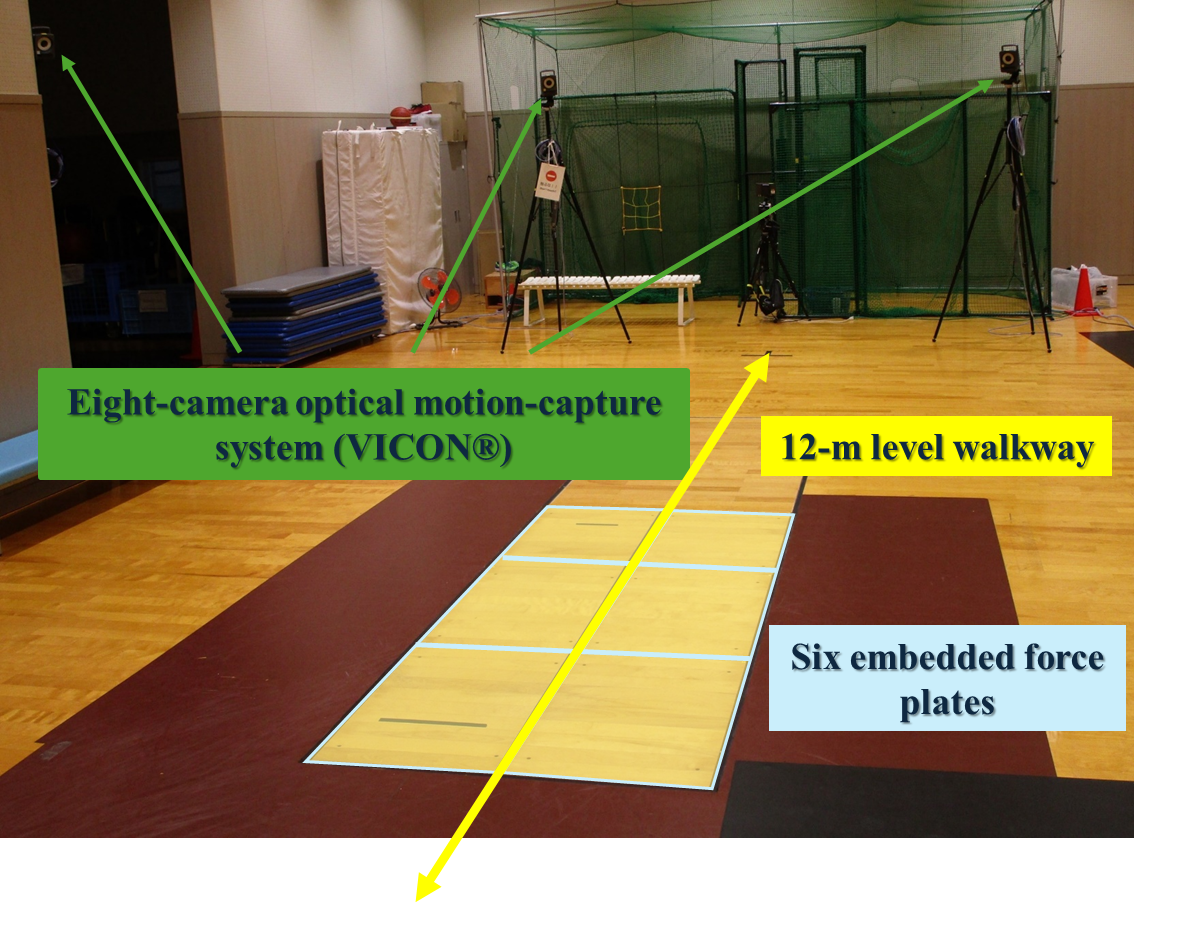

Supplement: Supplementary file 1 — Supplementary Figure 1. Experimental setup for image‐integrated motion analysis. (A) Optical motion‐capture system with eight infrared cameras and six force plates embedded in a 12‐m level walkway. (B) Representative lower‐limb marker placement during overground walking. Reflective markers were attached to the pelvis and lower limbs, and custom reflective markers incorporating radiopaque steel balls were attached to the thigh and shank to allow identification on subsequent biplanar radiographic images. [file JEO2-13-e70823-s001.png]

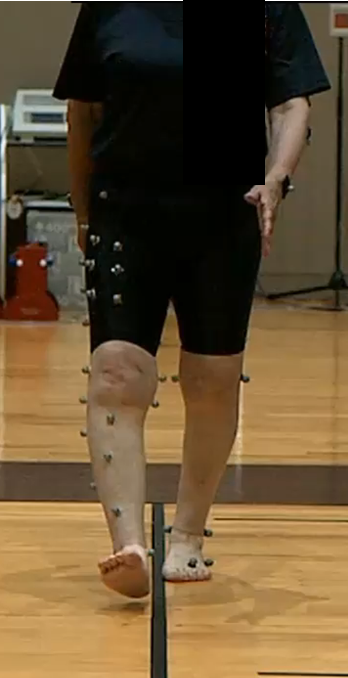

Supplement: Supplementary file 2 — Supporting File 1. [file JEO2-13-e70823-s002.png]
